# Plant phenolic volatiles inhibit quorum sensing in pectobacteria and reduce their virulence by potential binding to ExpI and ExpR proteins

**DOI:** 10.1038/srep38126

**Published:** 2016-12-01

**Authors:** Janak Raj Joshi, Netaly Khazanov, Hanoch Senderowitz, Saul Burdman, Alexander Lipsky, Iris Yedidia

**Affiliations:** 1 Department of Plant Pathology and Microbiology and the Otto Warburg Minerva Center for Agricultural Biotechnology, The Robert H. Smith Faculty of Agriculture, Food and Environment, The Hebrew University of Jerusalem, Rehovot, Israel; 2 Department of Plant Sciences, Agricultural Research Organization, The Volcani Center, Bet Dagan, Israel; 3 Department of Chemistry, Bar-Ilan University, Ramat-Gan, Israel

## Abstract

Quorum sensing (QS) is a population density-dependent regulatory system in bacteria that couples gene expression to cell density through accumulation of diffusible signaling molecules. Pectobacteria are causal agents of soft rot disease in a range of economically important crops. They rely on QS to coordinate their main virulence factor, production of plant cell wall degrading enzymes (PCWDEs). Plants have evolved an array of antimicrobial compounds to anticipate and cope with pathogens, of which essential oils (EOs) are widely recognized. Here, volatile EOs, carvacrol and eugenol, were shown to specifically interfere with QS, the master regulator of virulence in pectobacteria, resulting in strong inhibition of QS genes, biofilm formation and PCWDEs, thereby leading to impaired infection. Accumulation of the signal molecule N-acylhomoserine lactone declined upon treatment with EOs, suggesting direct interaction of EOs with either homoserine lactone synthase (ExpI) or with the regulatory protein (ExpR). Homology models of both proteins were constructed and docking simulations were performed to test the above hypotheses. The resulting binding modes and docking scores of carvacrol and eugenol support potential binding to ExpI/ExpR, with stronger interactions than previously known inhibitors of both proteins. The results demonstrate the potential involvement of phytochemicals in the control of *Pectobacterium*.

*Pectobacterium* species are Gram-negative phytopathogens belonging to the *Enterobacteriaceae* family. These pathogens cause soft rot in a wide range of food plants as well as ornamental crops. Pectobacteria are facultative anaerobic necrotrophs that were recently ranked within the top-ten “honorable” list of the most noxious plant pathogenic bacteria, mainly due to their negative impact on potato production both in the field and during storage worldwide^1^. The wide host range of pectobacteria includes 35% of all angiosperm plant orders^2^,^3^, while their virulence depends greatly on their ability to secrete plant cell wall degrading enzymes (PCWDEs) and to form biofilm^4^,^5^. These virulence determinants are known to be strictly under the control of quorum sensing (QS)^6^, a mechanism that is also responsible for the genus’ remarkable capability to coordinate brute force and stealth modes of action during infection in plants^7^. The coordination of virulence is mediated by *N-acyl-homoserine lactones* (AHLs) which are synthesized by *N-acyl homoserine lactone* synthase ExpI, and detected by the sensory protein ExpR (LuxI and LuxR homologues in pectobacteria, respectively)^8^,^9^, thus affecting the synthesis of PCWDEs^8^,^10^,^11^. Moreover, the ability of the genus to survive in different environments (plants, soil and water) as well as their tolerance to a large range of temperatures makes them a difficult target, with no effective control measures to date^12^.

An appealing control strategy against bacteria is based on the use of plant essential oils (EOs) which play an important role in the protection of plants against bacterial and fungal pathogens^13^. EOs comprise a broad range of antimicrobial phytochemicals that display potent activity against a variety of cellular targets in the bacterial membrane and cytoplasm^13^,^14^. Some of these volatiles were previously designated as potential inhibitors of the QS machinery in bacteria^15^,^16^. Two such compounds are carvacrol and eugenol, which are part of the terpenoid and phenylpropanoid pathways, respectively. Both were found to be inhibitors of biofilm formation and QS in bacteria as well as outer membrane disintegrating agents in Gram negative bacteria^17^,^18^. Specific inhibition of QS by these compounds was described for *Chromobacterium violaceum* and *Pseudomonas aeruginosa*^14^,^17^,^18^. Therefore, carvacrol and eugenol can be considered good candidates to interfere with QS and QS-related virulence factors in *Pectobacterium*.

Understanding the role of QS system in pectobacteria has become one of the most attractive aims for identifying targets for new drugs that may interfere with cell density and efficiently affect bacterial virulence and infection. Carvacrol and eugenol were shown to display a wide range of antibacterial, anticandidal and antifungal capabilities^14^,^19^,^20^. Interference with virulence in *Pectobacterium*, and more specifically with the emerging *P. aroidearum* and *P. carotovorum* subsp*. brasiliense* holds great potential for bacterial control during storage of potato and other tuber crops. Thus, the objectives of the current work were to elucidate the molecular mechanisms underpinning the antimicrobial activities of carvacrol and eugenol on these pathogens and to establish a potential mode of action. Major virulence determinants such as biofilm formation, secretion of PCWDEs and QS-related gene expression were studied following exposure to both compounds *in vitro*, while infection capabilities and compensation assays with the QS signaling molecule AHL were studied *in vivo* using potato tubers, cabbage and calla lily leaves as hosts. Finally, homology models of the QS proteins of *Pectobacterium* ExpI and ExpR were constructed, and docking of carvacrol and eugenol to their respective binding sites was conducted for the first time. The resulting binding modes highlight the importance of specific ligand-protein interactions, which could be used in the future for design of more active ExpI and ExpR blockers.

## Results

### Carvacrol and eugenol reduce biofilm formation

**Figure 1:**
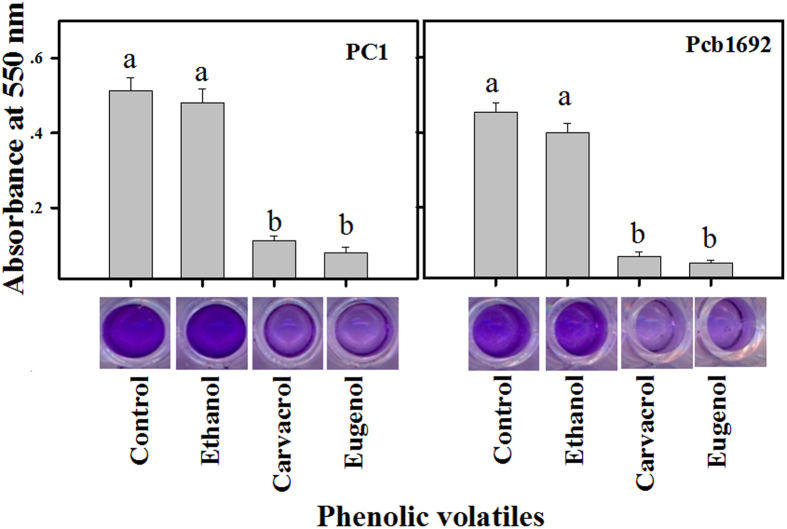
Carvacrol and eugenol reduce biofilm formation of *Pectobacterium aroidearum* PC1 and *Pectobacterium carotovorum* subsp. *brasiliense* Pcb1692. Biofilm formation was measured after 48 h of growth in LB medium, with or without 250 μM carvacrol or eugenol at 28 °C. Ethanol, the carrier of carvacrol and eugenol was used as an additional control along with LB medium control. Each bar represents the absorbance of crystal violet dye (bound to the biofilm cells) measured at 550 nm from two independent experiments. Treatments not connected by the same letters in each panel are significantly different from each other [*p* < 0.05; means ± standard errors (SE); n = 16].

A recent study demonstrated that *P. carotovorum* subsp. *brasiliense* colonizes the xylem tissue of symptomatic potatoes and forms biofilm-like aggregates that increase its capacity to remain in the infected host^21^. Here, we assessed the effect of the phenolic volatiles carvacrol and eugenol on the biofilm formation ability of two *Pectobacterium* strains, *P. carotovorum* subsp*. brasiliense* Pcb1692 and *P. aroidearum* PC1 (Table S1), using a microtiter dish assay with non-inhibitory concentrations (i.e. without causing growth inhibition) of these compounds. For both strains, a significant (*p* < 0.05) reduction in biofilm formation was observed at 250 μM of carvacrol and eugenol (Fig. 1). No such reduction was observed in the presence of ethanol, used as carrier of these volatiles, and the LB medium control treatment. Since carvacrol and eugenol dose-dependently inhibit the growth of *Pectobacterium* (Fig. S1), the concentrations of both volatiles for these assays were calibrated to a level that does not affect bacterial growth or membrane leakage (Fig. S2). Therefore, the observed reduction of biofilm formation in these assays was not a result of bacterial death or membrane disintegration.

### Carvacrol and eugenol halt secretion of PCWDEs

**Figure 2:**
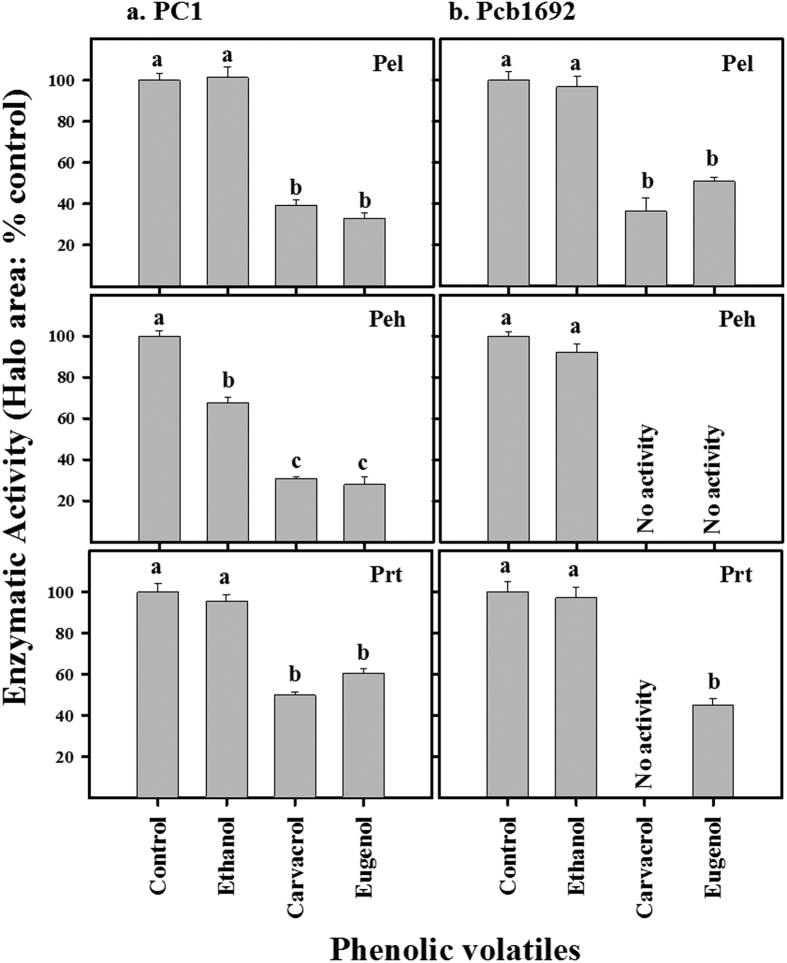
Carvacrol and eugenol reduce exoenzymes activities of (**a**) *P. aroidearum* PC1 and (**b**) *P. carotovorum* subsp. *brasiliense* Pcb1692. Enzymatic activity was evaluated following 8 h exposure of bacterial suspensions to nonlethal concentrations of the tested compounds (250 μM). Pectate lyase (Pel), polygalacturonase (Peh), and protease (Prt) activities were determined based on the size of substrate degradation haloes. Results are expressed as the percentage of activity relative to controls without the compounds. As an additional control treatment bacteria were exposed to ethanol that was used as carrier of carvacrol and eugenol. Each bar represents eight replicates, four from two independent experiments. Treatments not connected by the same letter in each panel are significantly different from each other (*p* < 0.05; means ± SE; n = 16).

PCWDEs are the primary arsenal of pectobacteria during soft rot maceration of the host tissue^5^,^22^. The activity of typical PCDWEs, i.e., pectate lyase (Pel), polygalacturonase (Peh) and protease (Prt) was assessed on strains PC1 and Pcb1692 in a semi-quantitative assay following exposure of the bacteria to non-inhibitory concentration of carvacrol and eugenol (250 μM). Overall, both compounds significantly (*p* < 0.05) reduced the activities of all tested PCWDEs in the two strains (Fig. 2). Moreover, both volatiles completely blocked the activity of Peh in strain Pcb1692, whereas carvacrol also blocked the activity of Prt in the same strain. In all combinations, ethanol had similar activity to that of LB alone, demonstrating that the observed inhibition effects in these assays resulted solely from carvacrol and eugenol.

### Carvacrol and eugenol reduce the expression of QS-related genes

**Figure 3:**
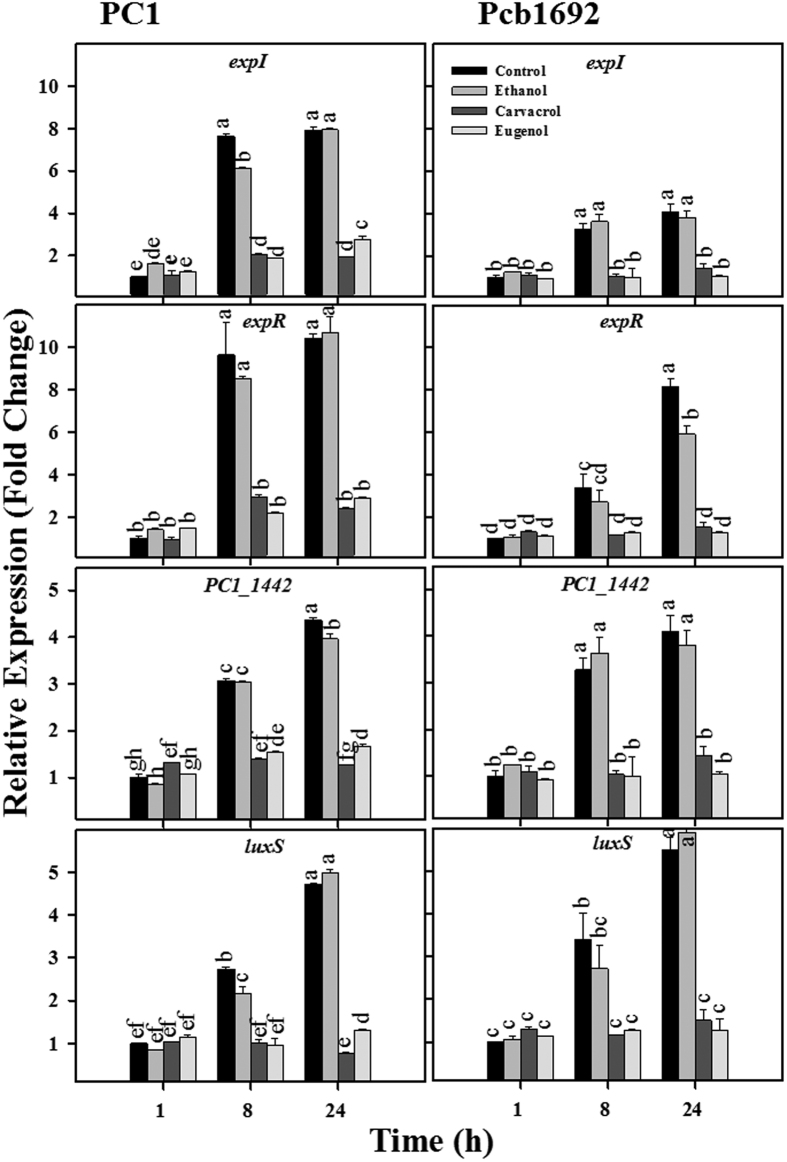
Effects of carvacrol and eugenol on transcript levels of quorum sensing (QS) system genes in *P. aroidearum* PC1 and *P. carotovorum* subsp. *brasiliense* Pcb1692. The transcript levels of the AI-1 QS system genes *expI, expR* and PC1_*1442 (luxR* transcription regulator) and the AI-2 QS system gene *luxS* were determined by quantitative real time-polymerase chain reaction (qRT-PCR) of cDNA samples prepared from RNA extracts of cultures grown in Lysogeny-Broth (LB) (28 °C, continuous shaking at 150 rpm) with or without (control) the phenolic volatiles at 250 μM. Ethanol, used as carrier of the compounds, served as an additional control. The data present the results from one representative experiment, out of two with similar results. Means ± SE of the relative expression of each gene (three replicates per treatment) are shown.

Biofilm formation and PCWDEs activity in pectobacteria are regulated by the QS system^7^. Accordingly, we further assessed the effects of carvacrol and eugenol on the expression of QS genes in strains PC1 and Pcb1692, by quantitative real time-PCR. As expected, in control or ethanol treatments the expression of genes *expI, expR* and *PC1_1442* (belonging to the AI-1 system) and *luxS* (belonging to the AI-2 system) increased with time in both strains. In contrast, during the same time period, both strains exposed to 250 μM carvacrol or eugenol did not display time-dependent increase in the expression of these genes (*p* < 0.05) (Fig. 3).

**Figure 4:**
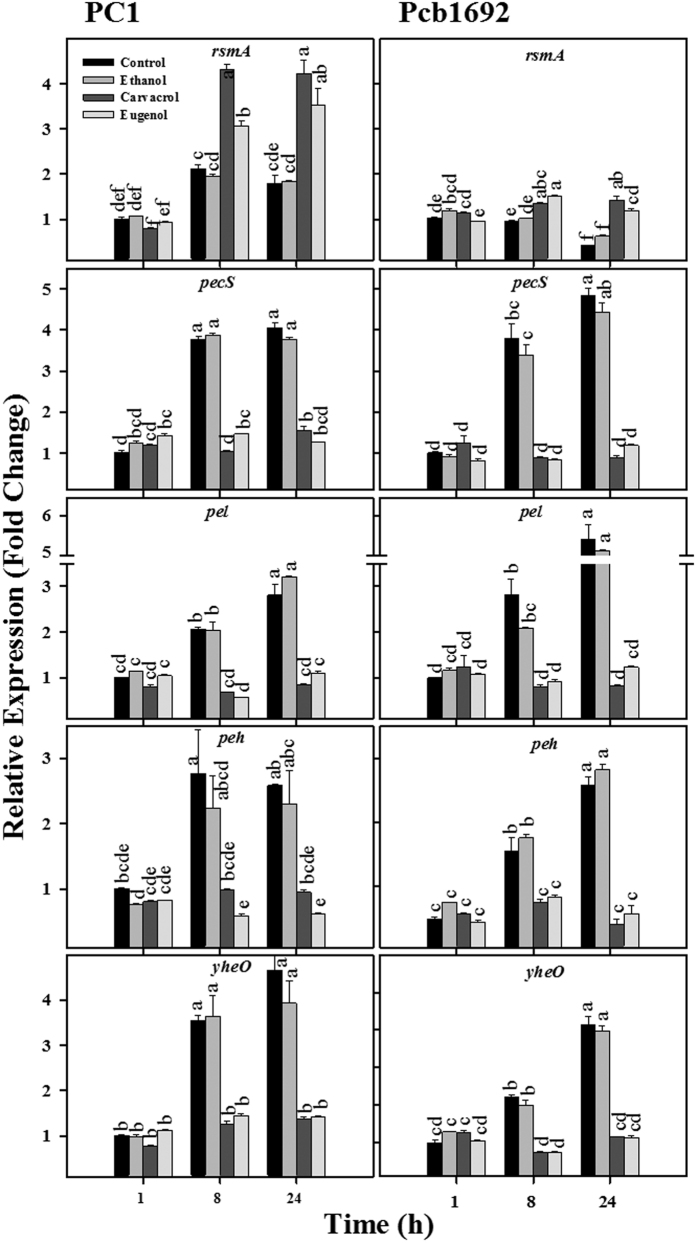
Effects of carvacrol and eugenol on transcript levels of quorum sensing (QS)-regulated genes in *P. aroidearum* PC1 and *P. carotovorum* subsp. *brasiliense* Pcb1692. The transcript levels of *rsmA, pecS, pel, peh* and *yheO* in RNA prepared from both bacterial species grown in Lysogeny-Broth (LB) medium (28 °C, continuous shaking at 150 rpm) with 250 μM carvacrol or eugenol or without these compounds (control) were determined by qRT-PCR. Ethanol, the carrier of the compounds, served as additional control. The data present the results from one representative experiment, out of two with similar results. Means ± SE of the relative expression of each gene (three replicates per treatment) are shown.

We also assessed the effects of carvacrol and eugenol on the expression levels of genes that are directly involved in the synthesis of PCWDEs. These genes are downstream of the QS cascade and are strictly controlled by the QS system^12^,^23^,^24^. Quantitative RT-PCR showed that the expression was suppressed. The relative expression of *rsmA*, a post transcriptional negative regulator of PCWDEs synthesis, increase in control and ethanol treated PC1 bacteria from 1 to 8 hrs, which is then constant from 8 hrs to 24 hrs. In Pcb1692, *rsmA* remained constant till 8 h and further reduced after 24 h under controlled condition and ethanol treatment. Similarly, in the presence of carvacrol or eugenol, the relative expression of *rsmA* in both strains was higher after 8 and 24 h relative to the controls with the strongest effects observed in strain PC1 (Fig. 4). In agreement with these findings, the relative expression of the PCWDEs genes *pecS, pel, peh* and *yheO* was clearly reduced by carvacrol and eugenol relative to control and ethanol treatments. In fact, the transcript levels of these genes did not rise with time in the presence of both phenolic volatiles (Fig. 4).

Furthermore, the effect of carvacrol and eugenol on the relative expression of genes *acrD* and *nssA*, encoding an efflux pump protein and a membrane transporter (assigned as “other genes” in Table S2) was assessed. These genes have been associated with virulence in pectobacteria, but their expression has not been reported to be directly associated with QS. In contrast with the clear effect observed on QS and QS-regulated genes, the expression of *acrD* and *nssA* genes was not affected by exposure to the volatiles over time (Fig. S3).

### Carvacrol and eugenol suppress the production of QS signaling molecules in *Pectobacterium*

**Figure 5:**
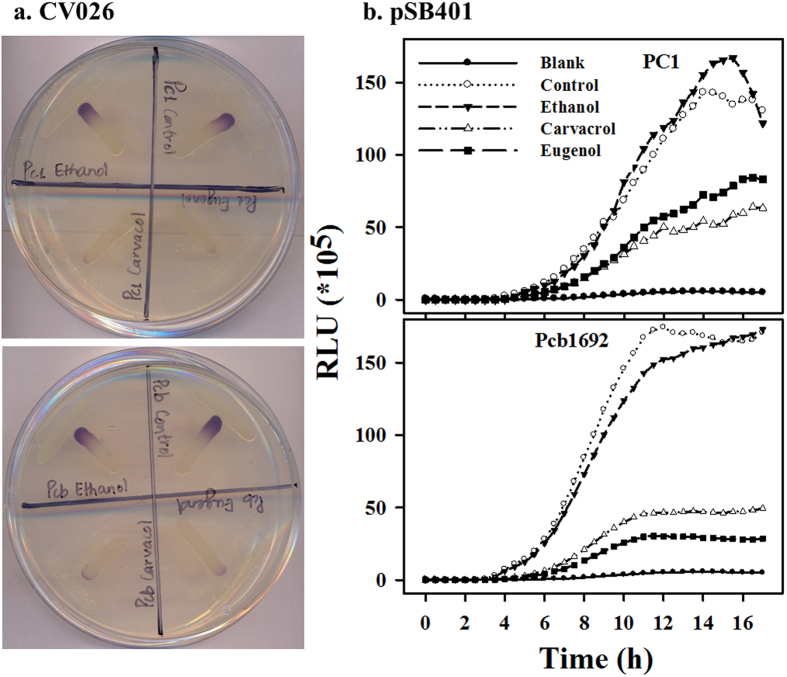
Carvacrol and eugenol reduce the production of quorum sensing (QS) signaling molecules by *P. aroidearum* PC1 and *P. carotovorum* subsp. *brasiliense* Pcb1692. (**a**) Violet pigment (violacein) formed by *Chromobacterium violaceum* CV026 in a response to N-acyl-homoserine lactones (AHLs) produced by PC1 (top) or Pcb1692 (bottom). Both bacterial strains were grown in the presence of non-inhibitory concentrations (250 μM) of carvacrol or eugenol or control treatments without the compounds. An additional control was exposure to ethanol, used as carrier of the volatile compounds. (**b**) Intensity of luminescence produced by *Escherichia coli* pSB401 induced by supernatants of strains PC1 (top) and Pcb1692 (bottom) grown with nonlethal concentrations (250 μM) of carvacrol, eugenol or control treatments without the compounds, or ethanol. Luminescence (250 ms) and absorbance (600 nm) were measured every 30 min for 17 h, and the relative luminescence (RLU = LU/OD_600nm_) was calculated. Blank was *E. coli* pSB401 grown in the absence of supernatants of *Pectobacterium* strains. Each data point represents means ± SE of eight replicates per treatment of one experiment, representative of two independent experiments with similar results. OD, optical density; LU, light units; RLU, relative light units.

AHLs are the known signaling molecules of the QS systems in *Pectobacterium* spp. that control the synthesis of PCWDEs^25^. To gain further insight into the mechanism of action of carvacrol and eugenol we assessed their effect on the production of AHLs, using reporter strains. The first qualitative assay was based on the reporter *Chromobacterium violaceum* CV026, which produces the purple pigment violacein in the presence of AHLs molecules^24^. Figure 5a clearly shows that PC1 and Pcb1692 cells exposed to carvacrol or eugenol induced lower levels of purple pigment in *C. violaceum* CV026 as compared to controls. In a second assay we used the *Escherichia coli* reporter strain pSB401, carrying the bioluminescence QS-reporter plasmid to quantitatively measure bioluminescence following exposure to the volatiles. Supernatants of bacterial cultures that were pre-exposed to either carvacrol or eugenol induced lower bioluminescence levels as compared to control or ethanol-treated cultures (Fig. 5b).

### Carvacrol and eugenol impairs virulence of *Pectobacterium* strains and treatment with exogenous AHL restores it

**Figure 6:**
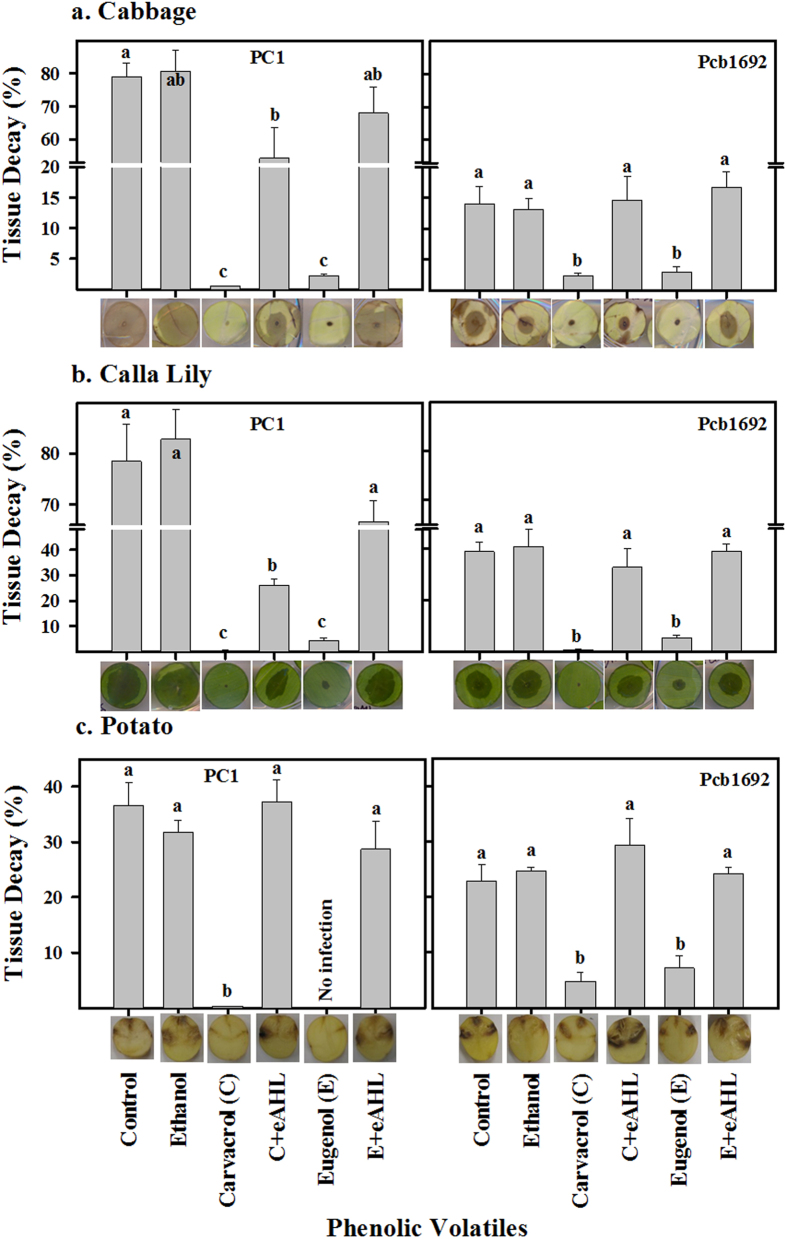
Carvacrol and eugenol impair virulence of *P. aroidearum* PC1 and *P. carotovorum* subsp. *brasiliense* Pcb1692 and exogenous application of eAHL restores it. Bacterial strains were exposed to carvacrol (C; 3 mM for PC1 and 1.5 mM for Pcb1692) or eugenol (E; 5 mM for PC1 and 3 mM for Pcb1692) before they were used to inoculate the plant tissue. Portions of the carvacrol- and eugenol-treated cultures were supplemented with ethanol (as the volatile carrier) and 100 nM of N-(β-ketocaproyl)-L-homoserine lactone (eAHL) before inoculation. Cabbage (**a**) and calla lily (**b**) leaf discs, and potato tubers (c) were injected with 10 μL of bacterial suspensions [10^6^ colony-forming units (CFU)] and incubated at 28 °C. Virulence was determined as the percentage of decayed tissue 24 and 48 h post inoculation of cabbage and calla lily leaf discs, and potato tubers, respectively, relative to the decay induced by untreated bacterial cultures (control). Data represent the means ± SE of 10 replicates for cabbage and calla lily and 4 replicates for potato from one experiment, representative of two independent experiments with similar results. Treatments that are not labeled with the same letter in each panel are significantly (*p* < 0.05) different. Representative photographs of infected discs of cabbage and calla lily, and potato tubers are shown for each treatment.

The inhibiting effects of carvacrol and eugenol on the activity of PCWDEs of strains PC1 and Pcb1692 suggested that these compounds may affect the virulence of these strains. To assess this question, virulence assays were conducted on three different hosts, cabbage, calla lily and potato, following exposure of both strains to carvacrol or eugenol alone, or in combination with N-(β-ketocaproyl)-L-homoserine lactone (eAHL) (Sigma Aldrich, UK). In the presence of carvacrol or eugenol, infection of the three hosts by PC1 or Pcb1692 was significantly (*p* < 0.05) reduced. In contrast, exogenous application of AHL to carvacrol- and eugenol-treated bacterial cultures, successfully restored the virulence of both strains in the three hosts, as shown by significant (*p* < 0.05) increases in soft-rot symptoms relative to strains treated with the volatile compounds. In fact, in most combinations, these treatments (combination of volatile compounds and exogenous eAHL) did not significantly differ from control treatments in terms of soft-rot symptom extent (Fig. 6). We also assessed the effect of external application of eAHL on expression of the central QS system genes *expI* and *expR*. These genes were entirely inhibited following treatment with carvacrol and eugenol (Fig. 3), but displayed similar levels of expression to those of control treatments upon exogenous application of eAHL (Fig. S4).

### Effect of carvacrol and eugenol on biosynthesis and sensing of AHL

To verify whether carvacrol and eugenol directly target the QS synthase and/or sensory protein, a pGEM-T plasmid with T7 promoter carrying the *expI* gene from *P. carotovorum* subsp*. brasiliense* was constructed and transformed into a QS negative strain of *E. coli*, DH5α. This strain does not contain any transcription factors, regulators and enhancers of the QS system. Transformed cells were treated with both EOs at concentrations ranging from 100 to 400 μM, and the level of growth and synthesized AHL were estimated. While growth was not affected by the EOs, reduced levels of AHL signal were observed as shown by lower intensities of violacein formation by the QS reporter strain *C. violaceum* CV026 (Fig. S5).

The plasmid expressing *expI* was also transformed into *E. coli* pSB401, which carries a bioluminescence reporter plasmid with the QS sensory protein gene (*luxR*). Since LuxR recognizes AHL produced by pectobacteria and the residues for AHL binding (Trp 62, Trp 66, Asp 79 and Ser 137) are conserved with ExpR, the results obtained in *E. coli* pSB401 can be applied to ExpR. Exposure of the transformed strain (expressing both *expI* and *luxR*) to carvacrol and eugenol resulted in lower levels of luminescence relative to controls without the compounds, in a dose-dependent response (Fig. S6). Finally, a concentration-dependent luminescence response was also observed in *E. coli* pSB401 (expressing solely *luxR*) upon application of increasing concentrations of carvacrol and eugenol supplemented with constant level of eAHL, suggesting competitive inhibition of the active site (Fig. S7). Collectively, these results strongly support a direct effect of carvacrol and eugenol on QS central proteins.

### Homology modeling of ExpR and ExpI

**Figure 7:**
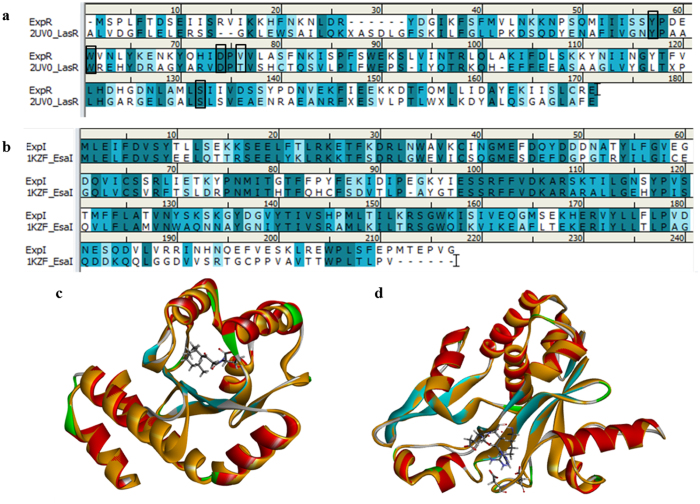
Sequence alignments and homology models for ExpR and ExpI. (**a**) Sequence alignment between LasR (2UV0) and ExpR. (**b**) Sequence alignment between EsaI (1KZF) and ExpI. Identical, similar and non-similar residues are colored in dark blue, light blue and white, respectively. (**c**) Superposition between ExpR and LasR. Conserved residues Tyr56, Trp60, Asp73 and Ser129 are shown in stick representation. (**d**) Superposition between ExpI and EsaI. The most important residues for the activity Asp45, Asp48, Arg68, Glu97, Ser99 and Arg100, are presented in stick representation. In (**c**,**d**), target structures are shown as ribbon diagram, color coded according to secondary structure and crystal structures are shown as ribbon diagrams colored in orange.

The aforementioned findings suggest that carvacrol and eugenol could directly interact with ExpI, ExpR or possibly other QS proteins to influence the QS machinery of pectobacteria. Since the crystal structure of the proteins are not available, homology models were created based on the crystal structures of the closest homologous with solved structures, LasR (LuxR homologue in *Pseudomonas aerugonisa*) and EsaI (LuxI homologue in *Pantoea stewarti*)^23^. Pair-wise sequence alignment resulted in 22.8% sequence identity between ExpR and its template, LasR (Fig. 7a), and 43.1% sequence identity between ExpI and its template, EsaI synthase (Fig. 7b). These values are above the 20% threshold considered to be appropriate for homology modeling^26^,^27^. Importantly four of the five residues (Tyr56, Trp60, Asp73, Thr75 and Ser129) that participate in H-bond interactions in the 3-oxo-C_12_-HSL (the LasR crystallographic ligand) binding site are conserved between ExpR and LasR (Fig. 7a; Thr75 is the non-conserved residue). The most important residues for the activity of EsaI (Asp45, Asp48, Arg68, Glu97, Ser99, and Arg100)^23^ are conserved in the sequence alignment between ExpI and EsaI (Fig. 7b).

Homology models based on these alignments were found to have good stereochemical qualities with 98.7% (ExpR) and 98.9% (ExpI) of residues residing in the most favored and additionally allowed regions of the Ramachndran plot and overall G-factors^28^ of −0.19 for ExpR and −0.17 for ExpI. Prosa profiles were similar to the templates with z-score of −6.72 for both models. Finally, template/target RMSD based on Cα atoms were 0.52 Å and 0.27 Å for ExpR (Fig. 7c) and ExpI (Fig. 7d), respectively.

### Docking of carvacrol and eugenol to the homology models of ExpR and ExpI

**Figure 8:**
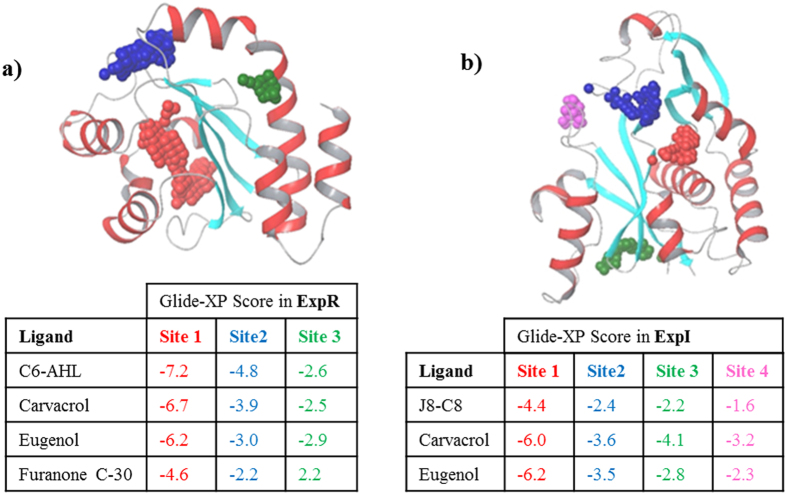
Docking of compounds into the three and four top-ranking cavities of the homology model of ExpR (**a**) and ExpI (**b**). (**a)** Docking scores of the compounds into the three top-ranking cavities (site 1: red, site 2: blue, site 3: green) in ExpR. The highest ranking site (red) corresponds to the binding site of the crystallographic ligand (3-oxo-C12-HSL, PDB code 2UV0). (**b)** Docking scores of the compounds into the four top-ranking cavities (site 1: red. site 2: blue, site 3: green. site 4: purple) in ExpI. Site 2 corresponds to the cavity that includes the residues found to be important for the enzymatic activity of EsaI (Asp45, Glu97, Ser 99 and Thr140).

The ExpR homology model was used for the docking of its natural ligand (C6-AHL), a known inhibitor of this protein (Furanone C-30)^25^,^29^, and the two compounds studied in this work, carvacrol and eugenol. Prior to docking three top-ranking binding sites were identified by SiteMap^30^ with the highest ranking cavity matching the known binding site of the crystallographic ligand (3-oxo-C12-HSL, PDB code 2UV0) (Fig. 8a). In this site, the highest ranking poses of C6-AHL forms four hydrogen bonds with binding site residues Tyr50, Trp54, Asp67 and Ser125 (Fig. S8). These residues correspond to Tyr56, Trp60, Asp73 and Ser129 in the crystal structure of LasR and are involved in similar interactions with 3-oxo-C12-HSL. Thus C6-AHL and 3-oxo-C12-HSL share a similar binding mode in their respective sites. Eugenol and carvacrol adopt very similar binding modes, with eugenol forming two hydrogen bonds with the side chain of Lys30 and the backbone of Pro41, and carvacrol forming one hydrogen bond with the side chain of Lys30. In contrast, the known inhibitor furanone C-30 adopts a different binding mode and forms one hydrogen bond with the side chain of Ser125 (Fig. S8). The GlideScore values of C6-AHL, eugenol, carvacol and furanone to this site are −7.2, −6.7, −6.2, and −4.6 kcal/mol, respectively, suggesting the natural ligand to be the strongest binder followed by the two compounds studied in this work. The known inhibitor furanone C-30 is the weakest binder, presumably due to its different binding mode. The docking scores of all ligands to the other sites are lower suggesting lower binding probability.

Three compounds carvacrol, eugenol and J8-C8 (a known inhibitor of the protein) were docked into the four-top-ranking cavities identified by SiteMap in the ExpI model(Fig. 8b)^31^,^32^. Site 2 (colored blue) to the cavity that includes the residues found to be important for the enzymatic activity of EsaI (Asp45, Glu97, Ser99 and Thr140)^23^. However, all compounds have better docking scores to the nearby located, higher scoring site 1 (colored red) which binds the acyl chain of the acyl-acyl carrier protein^23^. Both carvacrol and eugenol form Π-Π interactions with Phe123 while carvacrol also forms such interactions with Phe102. In contrast, J8-C8 forms two hydrogen bonds with Phe102 and Lys106 (correspond to Phe101 and Lys105 in EsaI) (Fig. S8). The GlideScore value of the more flexible ligand, J8-C8 is the lowest among the three compounds due to a larger penalty resulting from the freezing of rotatable bonds. The two other ligands, carvacrol and eugenol (with almost identical GlideScore values) are significantly less flexible.

For this protein we were unable to dock the natural ligand S-adenosyl- L-methionine (SAM). The SAM binding site, located at the highly conserved the N-terminus (residues Phe27, Arg30 and Trp33 in ExpI corresponding to residues Phe28, Asp31 and Trp34 in EsaI structure) was unresolved in the crystal structure of the EsaI template due to higher than average B factors. Interestingly these residues are well resolved in the related LasI structure^33^, yet this structure features a much lower sequence identity to the ExpI target and could not be used for homology modeling.

## Discussion

Soft rot enerobacterial plant pathogens of the genus *Pectobacterium* rely on QS for the production of an arsenal of plant cell wall degrading enzymes (PCWDEs). The important role of QS in the bacterial pathogenesis of many plants and animals has made this machinery an attractive target for bacterial control, and anti-QS compounds are an important focus of both basic and translational research. In this regard, carvacrol and eugenol, common components of EOs, were shown to exhibit antimicrobial activities against many Gram positive and Gram negative bacteria including *Pectobacterium*^14^. However, their specific effect on the QS system in bacteria was described previously only for *Chromobacterium violaceum* (carvacrol) and *Pseudomonas aeruginosa* (eugenol)^18^,^34^. Although both compounds were reported to have QS inhibiting activity, no mechanism of action was suggested for the interaction of these molecules with the QS central proteins.

In order to provide further insight into the specific mode of action of carvacrol and eugenol, in the present study we assessed their potential as inhibitors of the QS machinery in two *Pectobacterium* strains, *P. aroidearum* PC1and *P. carotovorum* subsp. *brasiliense* Pcb1692. Independent of their biosynthesis pathway, the mode of action of both carvacrol and eugenol seems to rely, at least in part, on the increase of outer membranes fluidity and permeability^14^. In line with previous reports on antimicrobial activity of carvacrol and eugenol^35^,^36^, we showed that these compounds exert dose-dependent effects in terms of toxicity and growth inhibition on both strains, PC1 and Pcb1692. Next, we determined non-inhibitory concentrations of these compounds that did not affect either membrane integrity or growth of these strains. By selecting these concentrations (250 μM for both compound/strain combinations) we could test whether these volatiles influence the QS machinery of the tested *Pectobacterium* strains. Our conclusions on potential mode of action of EOs were further corroborated by docking studies, which elucidated potential interactions between the volatiles and the central elements of the QS system in *Pectobacterium*, the ExpI and ExpR proteins.

Biofilm formation and PCWDEs synthesis are known to be tightly controlled by QS in several pathogens including *Pectobacterium* species^6^,^7^. Here we show that carvacrol and eugenol significantly reduced these activities in strains PC1 and Pcb1692. Reduced biofilm formation upon treatment with either carvacrol or eugenol was previously reported for *Chromobacterium violaceum, Pseudomonas aeruginosa* and *Candida albicans*, and in all cases was attributed to anti-QS activity or to membrane damage^18^,^34^,^37^,^38^. To further explore the mechanism of action of the compounds, their effect on expression of QS system genes was studied on AI-1(*expI, expR* and *PC1_1442*) and AI-2 (*luxS*) as well as on QS-regulated genes (*rsmA, pecS, pel, peh* and *YheO*) that contribute to the synthesis of PCWDEs. Our results demonstrated that carvacrol and eugenol significantly inhibited the expression of these genes in *P. aroidearum* PC1 and *P. carotovorum* subsp. *brasiliense* Pcb1692, from the exponential growth phase and onwards, relative to control treatments. These observations were strongly supported by a significant reduction of AHL accumulation in both strains following exposure to the compounds, as revealed by the use of two bacterial biosensor strains.

In the frame of this study we also assessed the effects of carvacrol and eugenol on expression of the *rsmA* gene, which encodes a negative regulator of PCWDEs synthesis^39^. According to the working model of QS regulation in *Pectobacterium* species, high levels of ExpI leads to a reduction of *rsmA* expression, which ultimately results in higher level of PCWDEs gene expression^8^,^39^,^40^. In agreement with this model, in our experiments we observed that under control conditions, *rsmA* displayed low expression patterns in both strains. In contrast, treatment with carvacrol or eugenol significantly increased *rsmA* expression at 8 and 24 h of growth in PC1, and at 24 h in Pcb1692, thus explaining the aforementioned reduced levels of PCWDEs gene expression in cells exposed to these volatiles. Inhibition of the QS major components (ExpI/ExpR) and PCWDEs gene expression upon treatment with carvacrol or eugenol was further reflected in lower enzymatic activities of the PCWDEs (pectate lyase, Pel; polygalacturonase, Peh and protease, Prt).

No study on anti-virulence function of active compounds is complete unless virulence assays are conducted and found to support the results. Here, the virulence capabilities of the two *Pectobacterium* strains were tested on three hosts, the monocot calla lily, and the dicots cabbage and potato. Exposure of the two *Pectobacterium* strains to carvacrol and eugenol significantly impaired their virulence and infection capabilities in the three tested hosts. Importantly, external supply of N-(β-ketocaproyl)-L-homoserine lactone (eAHL) to volatile-treated bacteria prior to infection restored bacterial virulence, in most cases, to control levels. Moreover, the expression levels of the QS system central genes *expI* and *expR*, which were completely inhibited by carvacrol and eugenol were almost fully recovered (Fig. S4). The effect of the two EOs was also tested in a QS negative strain of *E. coli*, DH5α, as well as in *E. coli* pSB401 expressing the QS sensory protein *luxR* attached to bioluminescence reporter plasmid. These strains were transformed with a plasmid carrying *P. carotovorum* subsp*. brasiliense expI*. Both EOs were shown to reduce the production of AHL molecule and to reduce the levels of QS-mediated luminescence in strains DH5α and *E. coli* pSB401 expressing the *expI* gene, respectively, thus supporting a direct effect of the compounds on the activity of the QS major proteins. Similar results using heterologous expression of QS proteins (LasI and RhlI) in *E. coli* were obtained upon application of tannic acid and trans-cinnamaldehyde by Chang *et al*.^41^, suggesting a more general role for plant derived phenolic compounds in the inhibition of QS. Collectively, the findings presented in this work indicate that carvacrol and eugenol interfere directly with the QS machinery in pectobacteria.

To further test this hypothesis at the atomic level, the potential interactions of carvacrol and eugenol with ExpI and ExpR were studied using docking tools. Carvacrol and eugenol, together with a few control compounds were docked into potential binding sites of ExpR and ExpI identified on homology models of both proteins. In both cases, eugenol and carvacrol scored better than the known inhibitors of ExpR and ExpI, furanone C-30 and J8-C8, respectively. For ExpR, the best docking scores were obtained for site 1 which is indeed the binding site of the crystallographic ligand (3-oxo-C12-HSL, PDB code 2UV0), supporting a direct competition between inhibitors and endogenous ligands. For ExpI the best docking scores were also obtained for site 1 while site 2 was implicated in endogenous ligand binding. Thus in this case we cannot exclude inhibition through binding to an allosteric site. Nevertheless these results strengthen the hypothesis by which these compounds indeed exert their effect by directly binding to ExpR and/or ExpI. Yet more work is clearly required in order to ascertain their exact mechanism of action. Understanding the molecular mechanism at the atomic level will undoubtedly facilitate the design of new inhibitors with improved anti-bacterial activity.

On the background of climate change, and unexpected soft rot bacteria outbreaks, aromatic volatiles that are defined safe by the US Food and Drug Administration (FDA 2006) hold great potential for soft rot control. The results of this work strongly support that the phenolic volatiles carvacrol and eugenol act through the QS machinery to inhibit specific virulence determinants in pectobacteria. The mechanism of both volatiles consists of direct inhibition of AHL production, potentially via direct interaction with ExpI/ExpR proteins. Further investigation is required to pinpoint the exact binding site(s) and mechanism of action.

## Methods

### Chemicals, bacteria and growth conditions

The purity of commercial carvacrol and eugenol (Sigma Aldrich, UK) used in this work was 98% and 99%, respectively. The stock solutions of both phenolic volatiles were prepared in 70% ethanol. Therefore, in all experiments we include a control treatment with the same volume of 70% ethanol. The bacterial strains used in this study are listed in Table S1. *Pectobacterium aroidearum* PC1, *Pectobacterium carotovorum* subsp. *brasiliense* Pcb1692 and *Chromobacterim violaceum* CV026 were cultivated at 28 °C; whereas *Escherichia coli* strains were cultivated at 37 °C. All strains were grown in Lysogeny-Broth (LB) medium (Difco Laboratories, USA) under continuous shaking (150 rpm) in a TU-400 incubator shaker. Murashiage and Skoog (MS) minimal medium (Duchefa, the Netherlands) was used for plant inoculation assays.

### Growth curves

*Pectobacterium* strains PC1 and Pcb1692 were grown overnight at 28 °C in 4 mL liquid LB medium under continuous shaking at 150 rpm. The suspensions were then diluted to a final concentration of 1 × 10^6^ CFU in 200 μL fresh LB and supplemented with carvacrol or eugenol, or without any compound (LB or LB with ethanol) for controls, in Bradford 96-well microtiter plates. Unless stated otherwise, the concentration of carvacrol and eugenol was 250 μM. Plates were incubated at 28 °C with continuous shaking, and growth was determined by measuring optical density at 600 nm (OD_600_) at 1 h intervals for 30 h, using a micro-plate reader.

### Biofilm Inhibition assay

The assay was performed using the microtiter dish assay with crystal violet (CV) for biofilm staining as described by O’Toolee, G. A.^42^. It was quantified by measuring absorbance at 550 nm in a micro-plate reader and represented as the absorbance of CV dye bound to biofilm cells. The mean of eight replicates were calculated after subtraction of the blank measurement.

### Activities of hydrolytic enzymes

The activities of pectate lyase (Pel), polygalacturonase (Peh) and proteolytic enzymes (Prt) were tested after 8 h exposure of bacteria grown in LB medium at 28 °C to non-inhibitory concentrations of carvacrol and eugenol. Semi-quantitative assays for Pel, Peh and Prt activities were performed using a plate assay as described by Chatterjee *et al*.^43^. The plates were prepared as described and then poked to form 4 mm holes, which were filled with the supernatants (25 μL) from overnight grown cultures and incubated at 28 °C for 18 h. Activity of the enzymes was expressed as the size of the observed haloes. Two independent experiments were carried out, each with eight replicates of each strain/compound combination.

### RNA extraction and cDNA preparation

*Pectobacterium* strains were grown overnight at 28 °C in LB medium under continuous shaking. Then, 1 mL from these cultures were centrifuged (7000 × g, 5 min) and transferred to 10 mL of fresh LB containing non-inhibitory concentrations of carvacrol or eugenol (250 μM). Controls (LB or LB + ethanol, ethanol used as carrier) were treated in the same manner. The fresh cultures were then grown at 28 °C under continuous shaking. Two-milliliter samples were collected at 1, 8 and 24 h (corresponding to lag, log and stationary phases, respectively; Fig. S2a) for RNA extraction, using the EZ-RNA II kit (Biological Industries, Israel). Extracted RNA was used to prepare cDNA, using the cDNA synthesis kit (Applied Biosystems, CA).

### Quantification of mRNA by quantitative real-time PCR (qRT-PCR)

We assessed the expression of pectobacterial virulence genes from three different categories, as described in Table S2: QS system genes, QS-regulated genes and others. Primer sequences already used in previous study were also used here to evaluate the level of transcripts for the selected genes^44^. QRT-PCR reaction mixtures contained 3.4 μL (17 ng) of cDNA, 5 μL of fast Syber green master mix and 0.8 μL (5 μM) of each forward and reverse primer. Reactions were performed using a Step One Plus Real-Time PCR system with the standard cycling parameters. The data were analyzed by the comparative C_T_ (ΔΔC_T_) method, with expression normalized to the expression of the reference gene *recA*.

### Qualitative assays for the detection of AHL molecules

Strain CV026 is a mini-Tn5 mutant of *Chromobacterium violaceum* in which the violet pigment violacein is induced in the presence of AHL compounds with N-acyl C_4_ to C_8_ side chains^24^. This assay was performed as a qualitative tool to assess the effect of carvacrol and eugenol on the production of AHLs in strains PC1 and Pcb1692. The *Pectobacterium* strains were grown in LB overnight as described above. Cultures were then centrifuged (7,000 × g, 5 min, at 28 °C) and bacterial pellets were re-suspended in fresh LB supplemented or not with non-inhibitory concentrations of carvacrol or eugenol. The reporter strain CV026 was grown in fresh LB medium supplemented with kanamycin (10 μg/mL). Then, CV026 and *Pectobacterium* strains were spread perpendicularly (in T-shape) on LB plates, with the reporter strain being spread a few millimeters away from the tested bacteria. The plates were incubated overnight at 28 °C and the intensity of violet color exhibited by the reporter strain was assessed.

### Quantitative assay for AHL molecules using a bioluminescence-based assay

*Escherichia coli* pSB401 is a bioluminescence-based QS biosensor, which was generated on the background of *E. coli* strain JM109. This strain carries the plasmid pSB401, which possesses the *luxRI’:luxCDABE (I*’means *luxI* mutated) bioluminescent reporter gene fusion^45^. This system can detect AHLs with acyl chains ranging from six to eight carbons in length (C_6_ to C_8_ AHLs)^46^. This strain was used to quantitatively assess the secretion of AHL molecules by *Pectobacterium* strains PC1 and Pcb1692 in the presence of carvacrol and eugenol. Overnight grown bacterial pellets of both *Pectobacterium* strains were suspended in fresh LB supplemented (or not) with non-inhibitory concentrations of carvacrol or eugenol and incubated for 8 h. Control and treated suspensions of PC1 and Pcb1692 were then centrifuged and 10 μL of supernatant was mixed with 190 μL of 5 × 10^6^ CFU/mL *E. coli* pSB401 in fresh LB medium in 96-well microtiter plates. The supernatant used (10 μL) contained carvacrol and eugenol and was diluted 20-folds, to a final volume of 200 μL in fresh LB. This 20-fold diluted concentration (12 μM) did not affect the growth and bioluminescence (on adding eAHL) of reporter strain psB401 (data not shown). Two hundred microliters of the reporter strain were used as a blank for the experiment and 1.5 μL of 70% ethanol (carrier of carvacrol and eugenol) were used as control. The plates were then incubated at 37 °C for 17 h. Bioluminescence and optical density were automatically and simultaneously determined using Enspire 2300 multilabel reader (PerkinElmer, USA), every 30 min at 250 ms and 600 nm, respectively. Bioluminescence was calculated as relative light units (RLU) per unit of optical density at 600 nm, which accounted for the influence of the different treatments on total bioluminescence.

### Virulence and compensation assays

Virulence and compensation assays were conducted by assessing symptom severity in three plants, *Brassica oleracea* (cabbage), *Zantedeschia aethiopica* (calla lily) and *Solanum tuberosum* (potato) ‘Lady Rosetta’. To explore the effect of eAHL, higher concentrations of carvacrol (3 mM for PC1 and 1.5 mM for Pcb1692) and eugenol (5 mM for PC1 and 3 mM for Pcb1692) were used in these experiments. These concentrations significantly reduced the development of visible symptoms within 24 h (for cabbage and calla lily) and 48 h (for potato tubers) post infection. Thereafter, those concentrations of both phenolic volatiles were added with eAHL (100 nM) to study the effect in soft rot symptoms. The experiment was performed on fully expanded young leaves of cabbage and calla lily and small (about 25–40 g) potato tubers as previously described^47^.

### Generation of a plasmid carrying the *expI* open reading frame

High-Fidelity DNA polymerase was used for PCR amplification of *expI* gene (without promoter) from *P. carotovorum* subsp. *brasiliense Pcb1692* following manufacturer’s instructions (NEB, USA). For *expI* gene, the forward and reverse primers used expIF (5′-CGAGCATATGTTAGAGATATTTGATGTAAATC-3′) and expIR (5′-GAACGGATCCTCAAGCCTGTGCAATAG-3′) (Hylabs, Israel). The PCR products were ligated into PGEMT-easy vector and the ligation mixture was electroporated into *E. coli* DH5α. The resulting plasmid was sequenced (Weizmann Institute, Israel) for the validation of insertion using T7 promoter. *E. coli* expressing LuxR protein attached to the bioluminescence plasmid (pSB401) was kindly provided by Dr. Leonid Chernin and Dr. Yael Helman. The LuxR protein can detect AHL produced by pectobacteria and also contains the conserved residues for AHL binding in pectobacteria, so the effect observed in LuxR was generalized to ExpR.

### Homology modeling

Homology models of ExpR (UniProtKB identifier C6DIG7) and ExpI (UniProtKB identifier P33882) were generated based on the crystal structures of the homologous proteins, LasR (PDB code: 2UV0)^48^ and EsaI (PDB code: 1KZF)^23^, respectively. Pair-wise sequence alignments were performed using the ClustalW tool as implemented in Discovery Studio (DS) version 4.1 with default settings. The resulting alignments were used as input to the MODELLER program^49^ as implemented in DS. The highest ranked models in both cases were submitted to a side-chain refinement protocol using the ChiRotor algorithm^50^ and subsequently verified for stereochemical quality using Procheck^51^ and Prosa^52^.

### Docking

Prior to docking, protein models were prepared using the prepare protein protocol as implemented in Maestro (Schrödinger, USA) to assign the correct protonation states to titrate-able residues. The structures of carvacrol, eugenol, C6-AHL, Furanone C-30, and J8-C8 were manually drawn, converted to 3D coordinates and minimized with the OPLS 2005 force field. The minimized structures were processed by Ligprep as implemented in Maestro, to assign correct protonation states at physiological pH. Prior to docking, potential druggable binding sites on both proteins were identified using SiteMap^30^. Docking was performed using Glide XP^53^. For ExpR, docking grids were centered on three potential sites including the one containing the crystallographic ligand (3-oxo-C12-HSL, PDB code 2UV0). For this site the lowest energy pose of 3-oxo-C12-HSL reproduced the crystal structure with an RMSD of 1.4 Å (data not shown). For ExpI, docking grids were centered on four potential binding sites including the one containing Ser99 (a key residue at the center of the active site of EsaI). For this site the docking box also included Asp45 and Glu97 which were shown to greatly affect enzymatic activity^31^. Binding affinities were evaluated by GlideScore.

## Additional Information

**How to cite this article**: Joshi, J. R. *et al*. Plant phenolic volatiles inhibit quorum sensing in Pectobacteria and reduce their virulence by potential binding to ExpI and ExpR proteins. *Sci. Rep.* **6**, 38126; doi: 10.1038/srep38126 (2016).

**Publisher's note:** Springer Nature remains neutral with regard to jurisdictional claims in published maps and institutional affiliations.

## Supplementary Material

Supplementary Information
