# Supplementary material for: Plant phenolic volatiles inhibit quorum sensing in pectobacteria and reduce their virulence by potential binding to ExpI and ExpR proteins
Source: Sci Rep. 2016 Dec 1;6:38126. doi: 10.1038/srep38126 (PMC5131480; doi:10.1038/srep38126)
Supplement: Supplementary Information [file srep38126-s1.pdf]

Plant phenolic volatiles inhibit quorum sensing in pectobacteria and reduce their virulence by potential binding to ExpI and ExpR proteins

Janak Raj Joshi[1, 2], Netaly Khazanov[3], Hanoch Senderowitz[3], Saul Burdman[1], Alexander Lipsky[2] and Iris Yedidia[2*]

[1] Department of Plant Pathology and Microbiology and the Otto Warburg Minerva Center for Agricultural Biotechnology, The Robert H. Smith Faculty of Agriculture, Food and Environment, The Hebrew University of Jerusalem, Rehovot, Israel;

[2] Department of Plant Sciences, Agricultural Research Organization, The Volcani Center, Bet Dagan, Israel;

[3]Department of Chemistry, Bar-Ilan University, Ramat-Gan, Israel.

***** irisy@volcani.agri.gov.il

Supporting Information

Table S1. Bacterial strains and plasmids used in this study.

**Bacterial strains**	**Description**	**Reference/Source**
Pectobacterium aroidearum (PC1)	Monocot strain isolated from *Ornithogalum dubium*, NCBI Accession no. PRJNA31289	[2]
Pectobacterium carotovorum subsp. *brasiliense* (Pcb1692)	Dicot strain isolated from *Solanum tuberosum*, NCBI Accession no. PRJNA31121	[2]
Chromobacterim violaceum (CV026)	Mini-Tn5 mutant derived from *C. violaceum* ATCC 31532 Hg^R, *cvil*::Tn5 *xyl*E, Kan^R, plus spontaneous Str^R. AHL (C_4- C_8) biosensor, produces violacein (pigment) only in the presence of eAHL.	[24]
Escherichia coli (pSB401)	*luxRluxl*' (*Photobacterium fischeri* [ATCC 7744])::*lux*CDABE (*Photorhabdus luminescens* [ATCC 29999]) fusion; pACYC184-derived, Tet^R, AHL bioluminescent biosensor	[45]
E. coli DH5α	*sup*E44 Δ*lac*U169 *(*Δ80*lac*ZΔM15*) hsd*R17 *recA1EnA1 gyr*A96 *thi-1 relA1*	Invitrogen
E. coli DH5α_*expI*	DH5α::PGEMT_*expI*:Amp^R	
E. coli pSB401_*expI*	pSB401::PGEMT_*expI*:Amp^R	
**Plasmid**		
pGEM-T Easy	Commercial cloning vector, lacZ, Amp^r	Promega Corp.

Hg^R, hygromycin-resistant; Kan^R, kanamycin-resistant; Str^R, streptomycin-resistant; Tet^R, tetracycline-resistant.

Table S2. Genes assessed in this study.

	Genes/ Gene ID	Description of function	References
QS (AI-1 system)	*expI* (8135146)	Quorum-sensing signal generator; acyl-homoserine lactone synthase	[54]
	expR (8135145)	Quorum-sensing transcriptional regulator	[54]
	PC1_1442 (8132381)	Transcriptional regulator of LuxR-like proteins	(NCBI)
QS AI-2	*luxS* (8134173)	S-ribosylhomocysteinase; quorum sensing autoinducer-2 production	[55]
Genes regulated by quorum-sensing	*rsmA* (8134182)	Controls the production of PCWDEs and secondary metabolites	[11]
	pecS (8133198)	Transcriptional regulator that controls the production of various virulence factors	[56]
	pel (8133114)	Synthesis of pectate lyase	(NCBI)
	peh (8131918)	Synthesis of Polygalacturonase.	(NCBI)
	PC1_2249 (*yheO*) (8133193)	*yheO* domain-containing protein; DNA-binding protein controlling the production of pectolytic enzymes	[57]
Other genes	*acrD* (8134910)	Multidrug efflux pump	(NCBI)
	nssA (8130937)	Putative sodium/sulfate symporter or membrane transport	(NCBI)

Gene IDs mentioned in table were taken from GenBank (NCBI).

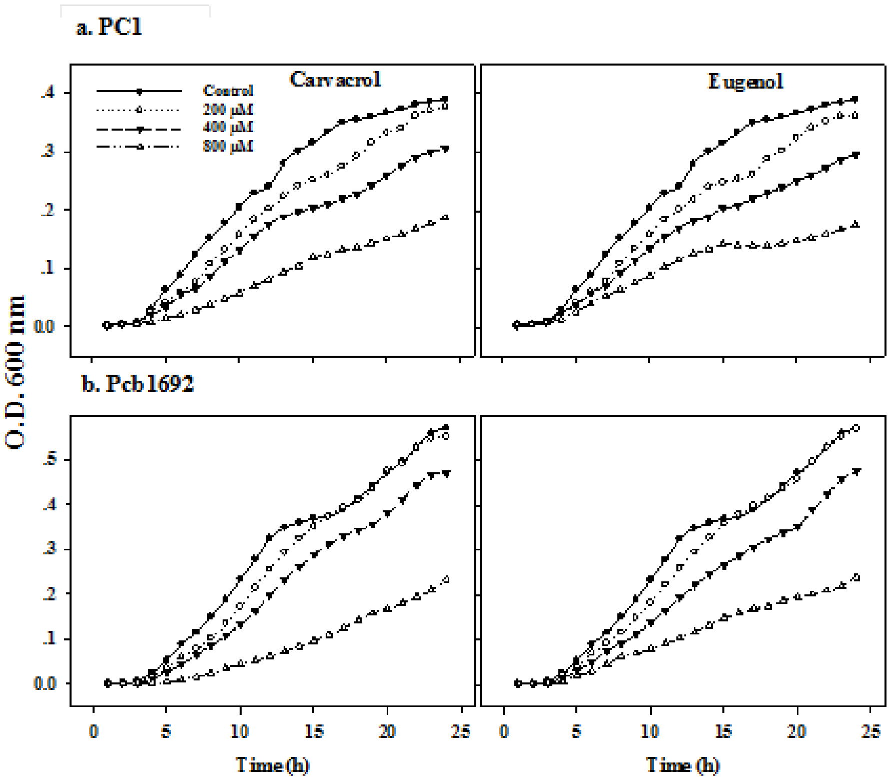

Figure S1. Dose-dependent effect of carvacrol and eugenol on growth of *Pectobacterium aroidearum* PC1 (A) and *Pectobacterium carotovorum* subsp *brasiliense* Pcb1692 (B). Both strains were grown at 28°C with continuous shaking in liquid LB (control) and LB with different concentrations of carvacrol (left) and eugenol (right). Absorbance (OD_{600}) was measured every hour, for 24 h,

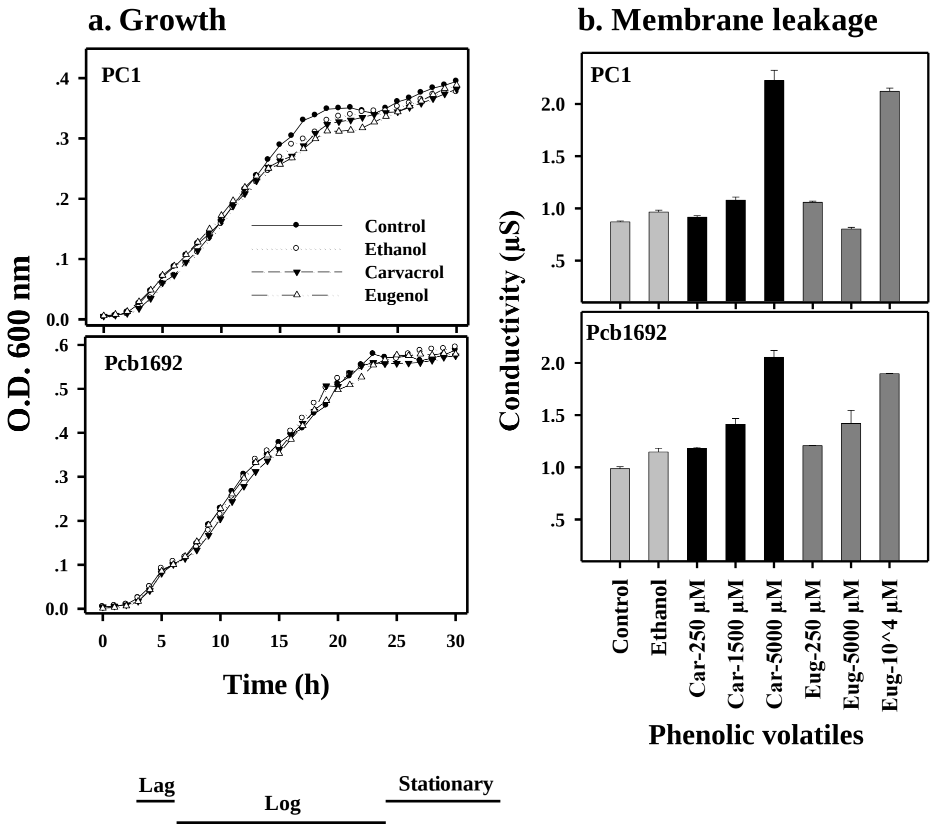

Figure S2. Effects of carvacrol and eugenol on growth and membrane leakage of *P. aroidearum* PC1 and *P. carotovorum* subsp *brasiliense* Pcb1692. (a) The strains were grown in liquid LB, LB with ethanol (that was used as carrier of carvacol and eugenol) and LB with (250 µM) of carvacrol or eugenol, with continuous shaking at 28°C. Absorbance (OD_{600}) was measured every hour for 30 h. (b) Overnight-grown bacteria were washed 3 times in sterile double distilled water (DDW) and suspended in DDW alone (control) or DDW containing ethanol or ethanol with carvacrol or eugenol at several concentrations. The conductivity was measured after 1 h.

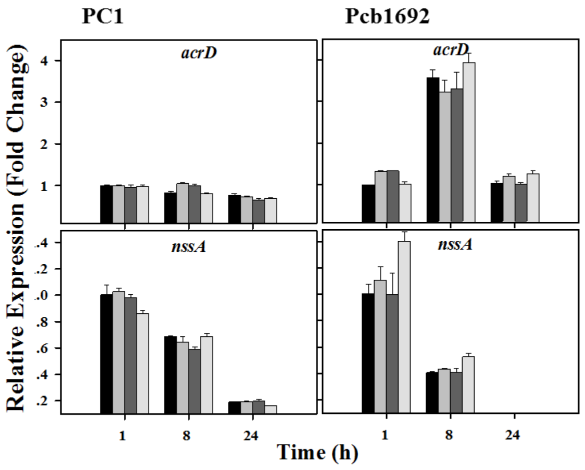

Figure S3. Effects of carvacrol and eugenol on expression of genes *acrD* and *nssA* of *P. aroidearum* PC1 and *P. carotovorum* subsp *brasiliense* Pcb1692. These genes contribute to bacterial virulence but are not known to be controlled by QS system.

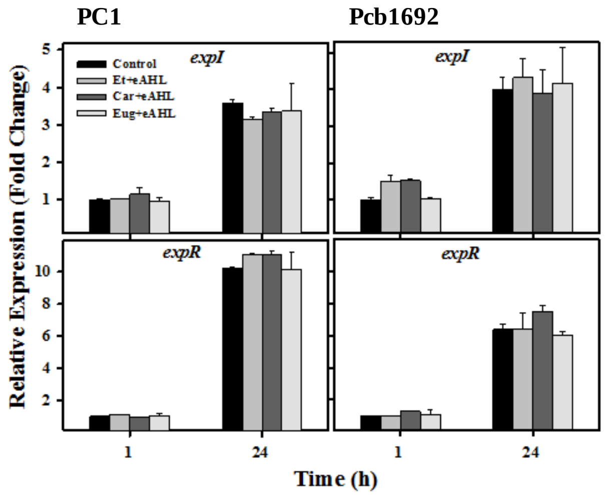

Figure S4. Restoration of the expression of quorum sensing (QS) system genes expI (AHL synthase) and expR (Receptor) by exogenous application of AHL in *P. aroidearum* PC1 and *P. carotovorum* subsp. *brasiliense* Pcb1692. Both *Pectobacterium* species were exposed to ethanol (Et, carrier) or carvacrol (Car; 3mM for PC1 and 1.5mM for Pcb1692) or eugenol (Eug; 5mM for PC1 and 3mM for Pcb1692) and supplemented with N-(β-ketocaproyl)-L-homoserine lactone (eAHL, 100nM) to check the expression level of *expI* and *expR* after 24 hours of treatment.

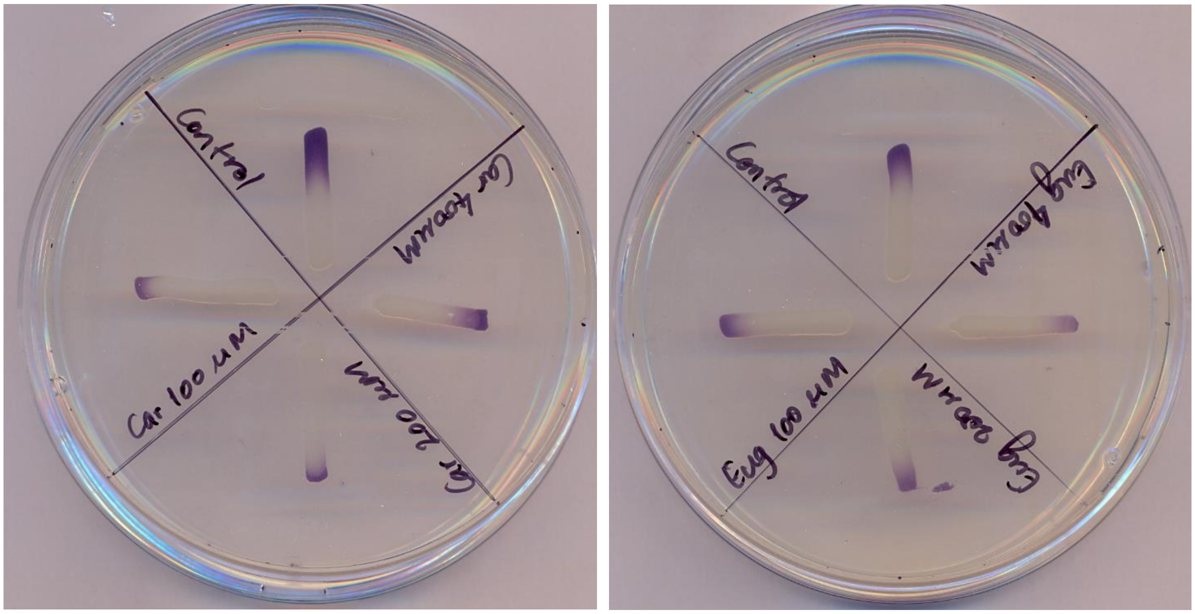

Figure S5. Carvacrol and eugenol reduce the production of quorum sensing (QS) signaling molecules produced by ExpI expressed in *E. coli* DH5α. Violet pigment (violacein) formed by *Chromobacterium violaceum* CV026 in response to N-acyl-homoserine lactones (AHLs), a QS signaling molecule. Transformed *E. coli* were grown in the presence of non-inhibitory concentrations (100-400 µM) of carvacrol (Car) or eugenol (Eug) or control treatments without the compounds. The reporter strain and the treated bacteria were streaked in T-shape (as shown in figure) in LB plate and incubated at28^{o}C for 15 h. The results presented are the representation of two independent experiments with similar outcomes.

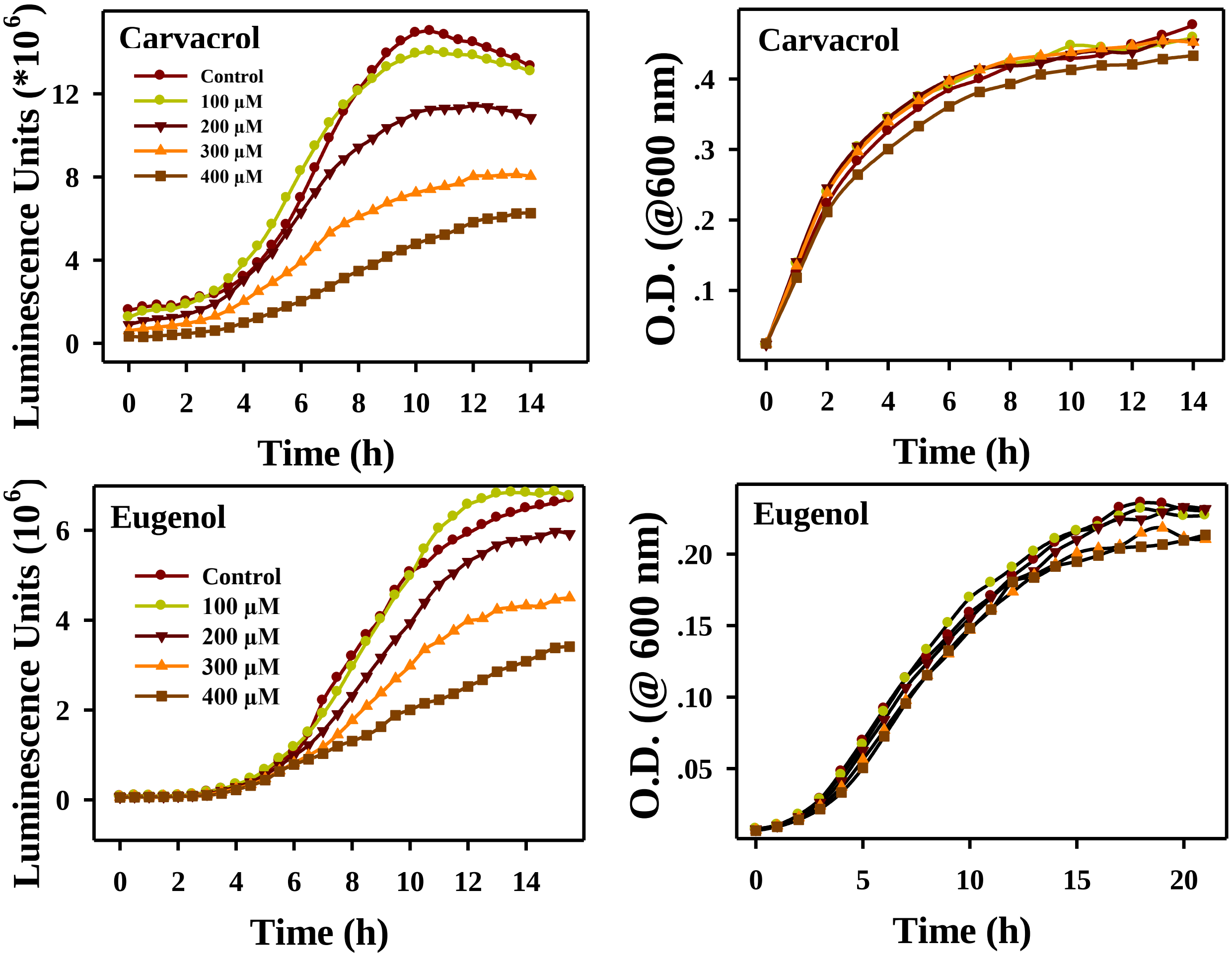

Figure S6. Carvacrol and eugenol reduce the production of luminescence signal produced by *E. coli* pSB401_expI. This strain contains a bioluminescence reporter plasmid carrying the QS sensory protein luxR and PGEMT vector containing the AHL synthase gene *expI* from *P. carotovorum* subsp. *brasiliense*. Bacteria were grown at 37°C in LB containing nonlethal concentrations (100-400 µM) of carvacrol or eugenol as well as without these compounds (control treatments). Luminescence (250 ms) and absorbance (600 nm) were measured to record the effect of the compounds on the QS system and growth, respectively. Each data point represents means of six replicates per treatment of one experiment, representative of two independent experiments with similar results.

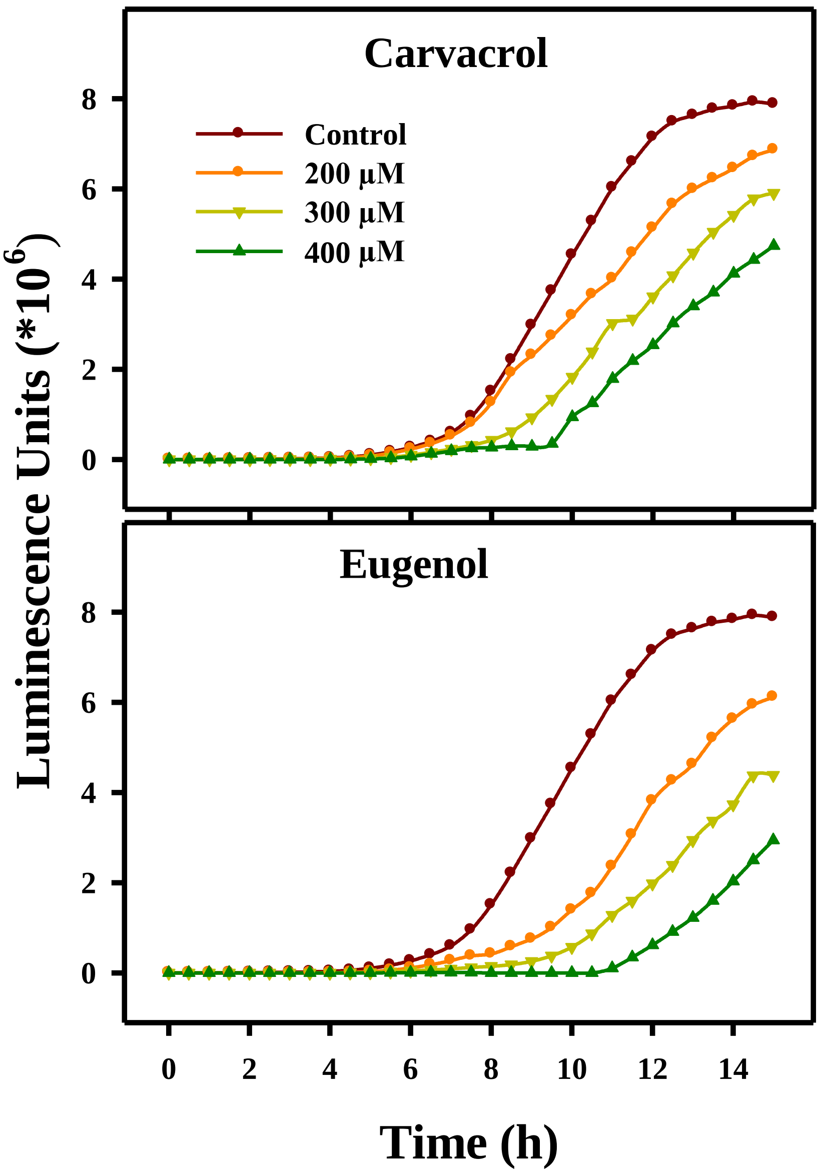

Figure S7. Carvacrol and eugenol reduce the production of luminescence signal produced by *E. coli* pSB401. This strain contains a bioluminescence reporter plasmid carrying the QS sensory protein *luxR* but mutated *luxI*. Luminescence signal are produced in presence of AHL supplied exogenously along with nonlethal concentrations (200-400 µM) of carvacrol or eugenol. Bacteria were grown at 37°C in LB containing as well as without the tested compounds (control treatments). Luminescence (250 ms) and absorbance (600 nm) were measured to record the effect of the compounds on LuxR and growth, respectively. Each data point represents means of six replicates per treatment of one experiment, representative of two independent experiments with similar results.

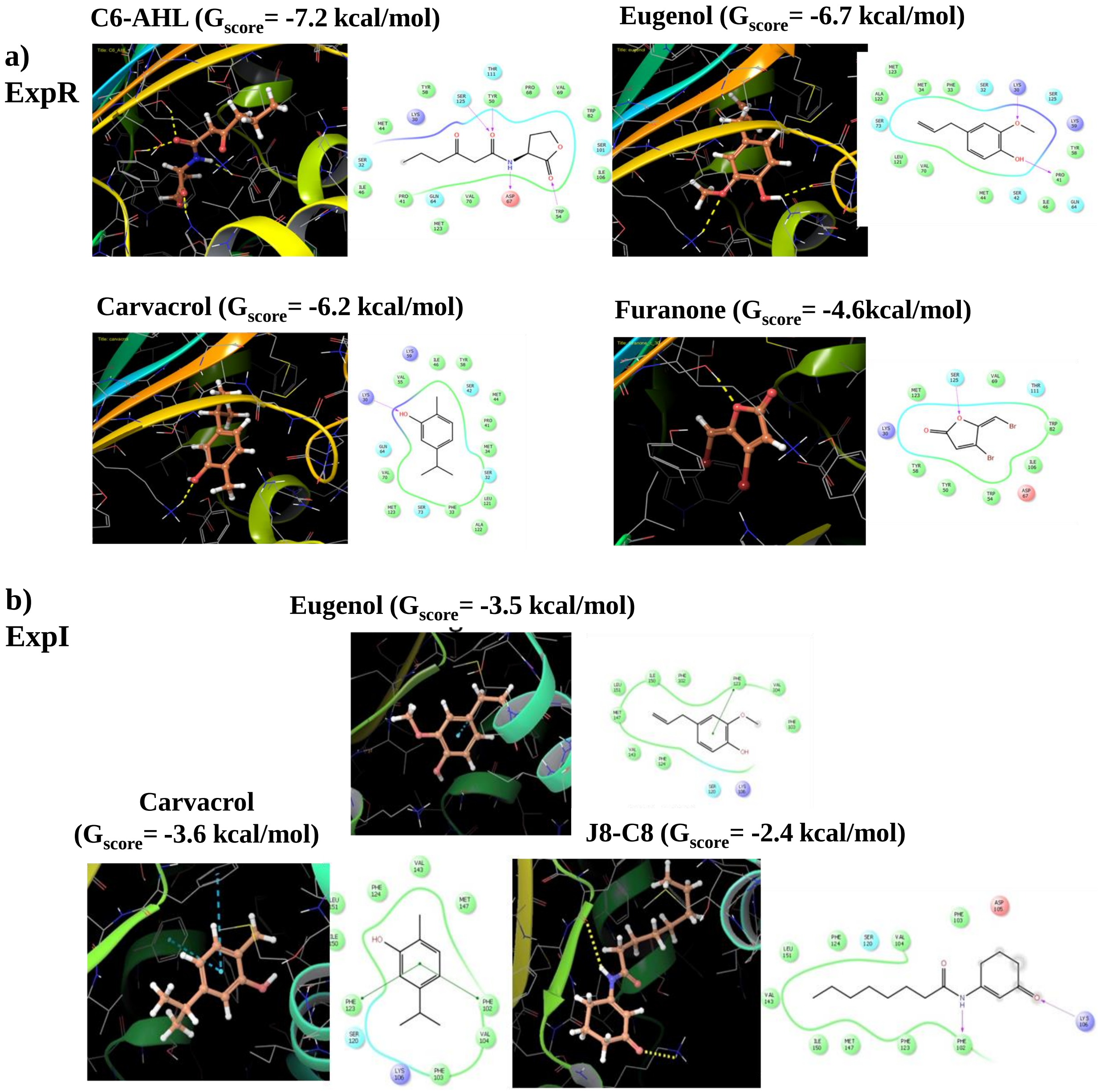

Figure S8. Ligand protein interactions. Three-dimensional (3D) and two-dimensional (2D) presentations of the interactions (within 4 Å from the docked compound) of the different compounds in: (a) site 1 (the site occupied by the crystallographic ligand) of ExpR and (b) site 1 of ExpI model. Red, blue, and green spheres represent positively charged, negatively charged and hydrophobic residues, respectively. Hydrogen bonds are presented as pink arrows in the 2D presentation and as yellow dashed lines in the 3D presentation. Π-Π stacking is shown as green lines in the 2D presentation and as cyan dashed lines in the 3D presentation.
